# Editorial: Rising Stars in Hematologic Malignancies 2021

**DOI:** 10.3389/fonc.2022.858564

**Published:** 2022-02-25

**Authors:** Liren Qian, Marcos De Lima, Alice S. Mims, Narendranath Epperla

**Affiliations:** ^1^ Senior Department of Hematology, The Fifth Medical Center of PLA General Hospital, Beijing, China; ^2^ Department of Medicine, Division of Hematology, The Ohio State University, Columbus, OH, United States

**Keywords:** hematologic malignancies, angioimmunoblastic T-cell lymphoma, graft-versus-host-disease, geriatric, chimeric antigen receptor

Although Harvey discovered the circulatory system as early as 1628, red blood cells were first described nearly 50 years later by Leeuwenhoek in 1674, and white blood cells nearly a century later by Hewson in 1770. Nearly 200 years later, the initial discovery of hematological malignancies occurred with the diagnosis of Hodgkin disease, first described by Hodgkin in 1832. This was followed by Virchow’s recognition of leukemia, the second hematological malignancy to be discovered (1–3). However, the past century is when major strides in our understanding of hematological malignancies occurred, especially in regard to advancements in pathobiology, diagnosis, and treatment. This is owed to rapid innovations in basic science, pharmacology, the invention of various advanced detection methods and instruments, the ability to more rapidly disseminate discovery through technology such as the internet, and the increased number of eminent scientists in the field. Cultivating an outstanding scientist is not easy, and requires many objective and subjective conditions. From the perspective of the general environment, the world needs relative peace. During World War I and II, medical progress stalled significantly, and the rapid development of hematology in the past half century has largely benefited from the relatively stable international environment. In terms of the local environment, it is necessary to have access to high-quality experimental platforms, facilities, and mentorship, along with excellent colleagues to improve our research through collaboration. There is also the significance for the ability to obtain scientific research funds to support and advance ongoing research. Most importantly, is that the researchers themselves needs to be proactive, creative, and driven. We aim with this Research Topiv to highlight and provide a platform for the ongoing research efforts for future rising stars in the field of hematological malignancies. Four up and coming investigators were selected across the entire breadth of Hematologic Malignancies, and within this special issue their publications present advances in theory, experiment, and methodology with applications to compelling problems. These four future thought leaders are ones to watch out for as they are likely to be major players in the continued shaping of the field of hematology.

In the article by Liu et al. from the Fifth Medical Center of PLA General Hospital, the authors report a novel approach for management of relapsed/refractory angioimmunoblastic T-cell lymphoma (AITL) treated by a new CAOLD chemotherapy regimen (cyclophosphamide, cytarabine, vindesine, pegaspargase, dexamethasone). Although there were only two patients with outcomes reported with this treatment regimen, the long-term survival data was impressive with one patient alive at over 5 years and the second over 7 years. More recently, novel agents such as brentuximab vedotin and checkpoint inhibitors have demonstrated activity in patients with AITL, but cytotoxic chemotherapy still remains the mainstay of treatment. Some questions remain unanswered such as if the CAOLD chemotherapy regimen can be safely combined with novel agents and improve the efficacy, could this therapy used as a bridge to allogeneic transplant, amongst other questions. Nevertheless, these findings are hypothesis-generating and this treatment regimens should be explored further in a large, multi-center clinical trial for relapsed/refractory AITL patients.

Snyder et al. from the Ohio State University reported a preclinical study demonstrated that bromodomain and extra-terminal domain (BET) inhibitors Plexxikon-51107/2853(PLX51107/2853) significantly alleviated graft-versus-host disease (GVHD) symptoms, prolonged survival overall, and increased survival rate without impairing graft-versus-leukemia effects in acute GVHD murine models by modulating the IL-23R/IL-17 axis. PLX51107 significantly improved survival rate in three murine models of acute GVHD. The survival rate increased nearly 70% in the CD45.1 B6 into B6D2F1 murine model, nearly 40% in the B6 into BALB/c murine model, about 50% in the C3S.W into B6 murine model. The survival rate increased more than 20% in the CD45.1 B6 into B6D2F1 model by PLX2853. Although Ruxolitinib, Vedolizumab, Tocilizumab, Infliximab, Brentuximab, anti-CCR5 monoclonal antibody, etc. have brought improvements to aGVHD in recent years, new drugs for aGVHD are still very important. These findings provide preclinical support for BET inhibitors in aGVHD, paving the way for clinical translational applications. Based on this research, the results of the authors phase 1 clinical trial are worth looking forward to.

Although geriatric assessments have been shown to be significant for guiding interventions in patients with hematological malignancies, the implementation of these assessments into current practice is infrequent. Wall et al. from the Ohio State University provided a multidimensional geriatric assessment to discriminate between different categories of patients with different prognosis. In the real-world study, increasing number of geriatric conditions and frailty were predictive of worse overall survival. The type of hematological malignancy (acute leukemia; myelodysplastic syndrome/myeloproliferative neoplasm/bone marrow failure), age (per 5-year increase), hemoglobin (per 1 g/dl decrease), deficit in activities of daily living, and Mini Nutrition Assessment score (at-risk of malnutrition vs. normal) were independent risk factors for prognosis. This study provides us with a guidance for how geriatric assessment and prescriptive intervention plan can and should be integrated into routine care of older adults with hematologic malignancies to improve outcomes.

In the review by Pasvolsky et al. from the University of Texas M.D. Anderson Cancer Center, the authors organized an overview of progress in immunotherapy for acute myeloid leukemia (AML), focusing on chimeric antigen receptor (CAR) technology to treat AML, highlighting the current state of CAR T cell therapy in AML, challenges and possible solutions for this therapy in AML, and future innovative directions in the field.

In conclusion, this Research Topic provides an excellent platform for the ongoing research for rising stars in hematological malignancies and continue to advance their careers.

Finally, we, guest editors, would like to thank the authors for their high-quality articles, the reviewers for their selfless contributions, and the editorial staff for their continued help and coordination.

## Author Contributions

All authors listed have made a substantial, direct, and intellectual contribution to the work and approved it for publication.

## Funding

LQ was supported by a grant from the National Defense Science and Technology Innovation Special Zone Project-Spark Project (Grant No. 20-163-00-TS-009-006-01) and a grant from the National Natural Science Foundation of China (Grant No. 81800180).

## Conflict of Interest

The authors declare that the research was conducted in the absence of any commercial or financial relationships that could be construed as a potential conflict of interest.

## Publisher’s Note

All claims expressed in this article are solely those of the authors and do not necessarily represent those of their affiliated organizations, or those of the publisher, the editors and the reviewers. Any product that may be evaluated in this article, or claim that may be made by its manufacturer, is not guaranteed or endorsed by the publisher.
